# The central executive network moderates the relationship between posttraumatic stress symptom severity and gastrointestinal related issues

**DOI:** 10.1038/s41598-024-61418-3

**Published:** 2024-05-10

**Authors:** Kia A. Howard, Salman S. Ahmad, Jennifer V. Chavez, Hannah Hoogerwoerd, Roger C. McIntosh

**Affiliations:** 1 Department of Psychology, University of Miami, Coral Gables, FL 33146 USA; 2 Department of Environmental Health Sciences, Robert Stempel College of Public Health & Social Work, Florida International University, Miami, FL 33199 USA

**Keywords:** Human behaviour, Stress and resilience

## Abstract

Although most adults experience at least one traumatic event in their lifetime, a smaller proportion will go on to be clinically diagnosed with post-traumatic stress disorder (PTSD). Persons diagnosed with PTSD have a greater likelihood of developing gastrointestinal (GI) disorders. However, the extent to which subclinical levels of post-traumatic stress (PTS) correspond with the incidence of GI issues in a normative sample is unclear. Resting state fMRI, medical history, psychological survey, and anthropometric data were acquired from the Enhanced Nathan Kline Institute-Rockland Sample (n = 378; age range 18–85.6 years). The primary aim of this study was to test the main effect of subclinical PTS symptom severity on the number of endorsed GI issues. The secondary aim was to test the moderating effect of high versus low resting state functional connectivity (rsFC) of the central executive network (CEN) on the relationship between PTS symptom severity and GI issues. Trauma Symptom Checklist-40 (TSC-40) scores were positively associated with the number of endorsed GI issues (b = −0.038, SE = .009, *p* < .001). The interaction between TSC-40 scores and rsFC within the CEN was significant on GI issues after controlling for sociodemographic and cardiometabolic variables (b = −0.031, SE = .016, *p* < .05), such that above average rsFC within the CEN buffered the effect of TSC-40 scores on GI issues. Our findings of higher rsFC within the CEN moderating the magnitude of coincidence in PTS and GI symptom severity may reflect the mitigating role of executive control processes in the putative stress signaling mechanisms that contribute to gut dysbiosis.

Over the past two decades, interest in the bidirectional communication between the central nervous (CNS) and gastrointestinal (GI) systems has peaked. Of particular interest is the connection between the pathophysiological processes involving GI disease and the CNS’s response to traumatic or stressful life events^1^. Current stress-diathesis models examining these effects imply that individuals with past trauma are at greater risk for developing GI dysregulation^2–4^. When exposed to a perceived traumatic or stressful life event, the CNS communicates with the GI system through stress hormones (e.g., cortisol, adrenaline, and norepinephrine). As an example, prolonged exposure to stress hormones can result in an imbalance in the gut’s microbiome (e.g., by altering the balance of beneficial versus harmful bacteria) and increase the permeability of the gut lining (known as “leaky gut”), leading to inflammation and reduced immune function that contribute to further GI dysfunctions^5^. Although outside the scope of the study, several other mechanisms have been implicated in the relationship between stress allostasis and gut dysbiosis^6–9^. In general, the complex interplay between stress hormones, microbiota composition, and immune response via the “gut-brain axis” allows us to examine the co-occurrence of GI disease in chronic stress conditions and probe the extent to which neural networks governing self-regulation may mitigate the stress-related GI pathophysiology.

Post-traumatic stress disorder (PTSD) is a type of chronic stress disorder that manifests at a clinical level following an exposure to or witnessing a event that is intensely frightening, shocking, or life-threatening, often involving scenarios of serious injury or mortal danger^10^. The temporary increase in post-traumatic stress (PTS) symptoms (such as arousal, fear, nightmares, dissociation, and avoidance of feared situations) following a traumatic event is considered a typical response, particularly when these symptoms gradually diminish with time despite continued exposure to trauma reminders^10,11^. However, those who experience symptoms lasting over a month may meet DSM-5-TR criteria for PTSD^12^ criteria for PTSD. Although 50–70% of U.S. adults experience at least one traumatic event in their lifetime, only 5–10% of these individuals go on to develop PTSD^12,13^. This begs the question of whether or not the putative neurobiological mechanisms underpinning stress-related gut dysbiosis are applicable to subclinical conditions and further necessitates an examination amongst individuals not meeting DSM-IV criteria for PTSD. This necessitates an examination into how subclinical and clinical experiences of trauma alike contribute to the intricate relationship between psychological stress and gastrointestinal health.

A meta-analysis of 71 studies comparing individuals with clinical GI disturbances, including Irritable Bowel Syndrome (IBS), to control/comparison groups determined that psychological trauma in both clinical and non-clinical samples was linked to a multitude of functional somatic syndromes^14^. Specifically, Afari et al.^14^ found that endorsements of trauma were associated with a 2.22 times greater likelihood of developing IBS. Another meta-analysis^15^ also found that endorsements of sexual abuse were associated with a greater likelihood of developing functional GI disorders for rape survivors. Various mechanisms have been proposed to explain how trauma-related psychological stress leads to the development of dysbiosis within the gut^16–18^. Due to their focus on individuals with PTSD, a comprehensive evaluation of these mechanisms linking the general experience of trauma to gut dysbiosis remains elusive. However, subclinical indicators of PTS, such as the Trauma Symptom Checklist-40 (TSC-40;^11,19^), have become helpful in refining our understanding of normative samples. For example, severe IBS symptomology has been shown to predict PTS symptoms, particularly in individuals who have experienced childhood trauma (emotional, physical, and sexual abuse)^20^.

Ample evidence supports the involvement of a consorted number of frontal lobe processes in the manifestation of and treatment response to PTSD. Individuals with PTSD are more likely to demonstrate weaker performance on measures of attention and working memory when compared to both trauma-exposed individuals and non-trauma controls^21^. Roelofs and Spinhoven^18^ reviewed what they described as the leading models that explain the relationship between trauma and medically unexplained symptoms, and proposed frontal lobe functioning as a key factor in the assimilation and regulation of affective processes that manifest in PTSD. Indeed, executive or frontal lobe function appears to be compromised in individuals diagnosed with PTSD^22–24^. Moreover, executive dysfunction or decline has been linked to the exacerbation of PTS symptoms over time^25^. Given the inherent limitations of the current assessment of neuropsychological functioning in the executive domain, attention has shifted to the neural underpinnings of executive control. Resting-state functional connectivity (rsFC) of the frontoparietal and subcortical brain regions predicts executive control in adults^26,27^.

In particular, the central executive network (CEN) connects areas of the dorsolateral prefrontal cortex and posterior partial cortices to support higher-order cognitive processes, including regulating emotions, behavior, and attention control^28,29^. Although not a population of focus of the current study, adolescent girls with PTSD exhibit weakened intrinsic connectivity of the CEN when compared to trauma-exposed controls. Moreover, CEN connectivity was also found to moderate trauma symptom severity^30^. In another study, decreased CEN connectivity was also associated with severity of PTSD^31^. However, most of the studies relating CEN to trauma have been conducted on clinical (i.e., PTSD) populations.

Despite this convincing evidence, there is a gap in the current literature concerning the moderating effect of CEN connectivity on the effect of trauma symptom severity on GI issues in trauma-exposed individuals with subthreshold PTSD symptomology. A potential moderating role of CEN connectivity on the co-incidence between trauma symptom burden and GI disease has been implied in previous research. For example, a recent study^32^ examined differences in rsFC among individuals with ulcerative colitis (UC, an inflammatory bowel disease that causes inflammation and ulcers in the digestive tract), IBS, and a healthy control group. The group with the most clinically severe GI issues (UC) reported the most severe psychological symptoms (e.g., increased anxiety scores) as well as reduced rsFC and reduced centrality of regions within the CEN. In this context, the reduced eigenvector centrality indicates decreased rsFC between the primary hubs of the CEN, which may lead to interference with cognitive control and executive function processes. The authors suggest that the connectivity of frontal brain regions supporting executive control functions less efficiently and is under higher metabolic costs in persons with more severe GI issues compared to the other groups, giving insight into the interdependencies of PTS, GI issues, and functional brain connectivity. However, the extent to which these relationships exist in persons experiencing subclinical levels of PTS remains unclear.

As such, our objective in this study is to broaden the scope of research to encompass subclinical populations experiencing PTS symptoms, while acknowledging that the existing body of literature is predominantly focused on clinical PTSD populations. Thus, we will leverage this substantial foundation to inform and guide the specific aims of our study.

## Methods

### Participants

The fMRI and physiological data were acquired from the Enhanced Nathan Kline Institute-Rockland Sample (NKI-RS)^33^. Institutional Review Board (IRB) approval was secured for the parent study from both the Nathan Kline Institute (Phase I #226781 and Phase II #239708) and Montclair State University (Phase I #000983A and Phase II #000983B). All participants provided written informed consent. Data obtained were processed and shared in strict accordance with Health Insurance Portability and Accountability Act (HIPAA) standards to guarantee participant anonymity. The data sharing protocol employed the removal of all potential HIPAA identifiers and the anonymization of facial features from anatomical images. Furthermore, this study adhered to a data use agreement of the Enhanced NKI-RS's data-sharing policy.

Data collection involved a semi-structured diagnostic psychiatric interview, a battery of psychiatric, cognitive, and behavioral assessments, and a multimodal brain imaging session described in previous studies^33^. Individuals with complete demographic, psychophysiological, rs-fMRI, and behavioral data were included in the analysis. Exclusionary criteria were based on a standardized clinical intake. They included a history of epilepsy, major depression, bipolar disorder, Alzheimer’s dementia, Huntington’s disease, meningitis, Parkinson’s disease, schizophrenia, general anxiety, and loss of consciousness following head injury. Furthermore, individuals with a previous or current diagnosis of PTSD (as determined via semi-structured diagnostic interviews) or any eating disorders were excluded from our analyses due to their confounding effects on GI function^34,35^. Participants were asked to report if they were ever diagnosed with, IBS, Crohn’s disease, UC, gastric reflux, and/or stomach/intestinal ulcers. In addition, participants were asked to self-report if they had experienced GI issues more generally. Those who endorsed general GI issues are reflected in the “stomach/intestinal problems” category in Table 1.

**Table 1:** Comparison of demographics by CEN Group Status.

	Low CEN rsFC (n = 202)	High CEN rsFC (n = 176)	T-statistic
TSC-40 scores	20.52 ± 12.45	17.36 ± 12.44	2.46*
GI Count	0.69 ± 1.06	0.52 ± 0.84	1.74
Age (years)	51.47 ± 17.20	45.62 ± 18.39	3.18*
Race (%)			−3.19*^a^
Black	14.36	24.43	
White	77.23	65.34	
Native American	0.5	0.57	
Asian	4.46	8.52	
Other	3.47	1.14	
Medical history (%)			
Stomach/intestinal problems	28.21	23.86	−0.96^a^
Irritable bowel syndrome	13.86	7.39	−2.02*^a^
Chron’s disease	0.5	1.14	−0.70^a^
Ulcerative colitis	1.98	1.14	−0.65^a^
Gastric reflux	18.81	14.77	−1.04^a^
Stomach/intestinal ulcers	5.94	3.98	−0.87^a^
Cardiometabolic factors			
Glucose	68.67 ± 47.82	72.14 ± 36.97	−0.79
Triglycerides	82.06 ± 124.15	82.78 ± 70.35	−0.07
HDL	48.06 ± 32.94	48.74 ± 28.17	−0.22
LDL	83.33 ± 58.82	90.32 ± 55.38	−1.19

^a^Mann-Whitney U Test was utilized for non-parametric comparisons of categorical data.

* *p* < .05.

Participants’ ethnicity/race was described as White (71.8%), Black or African American (19%), Asian (6.3%), Native American or Native Alaskan (0.5%), and other (2.4%). Of those who endorsed experiencing GI-related issues, general stomach/intestinal problems (26.1%), gastric reflux (16.9%), IBS (10.8%), stomach/intestinal ulcers (5%), UC (1.6%), and Crohn’s Disease (0.8%) made up the majority of their medical history.

### Measures

Symptoms related to stress and traumatic experiences were measured using the TSC-40. This scale was designed for the measurement of post-traumatic symptomatology associated with childhood trauma. The TSC-40 is a self-reported scale containing 40 items with six subscales: dissociation, anxiety, depression, a sexual abuse trauma index, sexual problems, and sleep disturbances^11^. Higher total TSC-40 scores signify higher trauma symptom severity. Construct validation for the total TSC-40 scale suggests that the scale demonstrates strong measurement invariance across participants with or without abuse-related and multiple trauma histories^36^. The TSC-40 has been used to assess PTS in several non-clinical samples^37^^11^. Cronbach’s alpha of the TSC-40 for the entire cohort was high, α = 0.898. Additionally, studies using the TSC-40 indicate that it is a relatively reliable measure (subscale alphas typically range from 0.66 to 0.77, with alphas for the full-scale averaging between 0.89 and 0.91)^38^.

### Cardiometabolic Variables

Body Mass Index (BMI) was calculated using reports of height (meters) and weight (kilograms) using the following formula: BMI = weight (kg)/height^2^ (m^2^). A complete metabolic panel was performed on fasting whole blood and included total serum cholesterol with a reference range for total serum cholesterol of 144–199 mg/dL. High-density lipoprotein (HDL) was also measured within a reference range of 35–70 mg/dL. The plasma lipid concentration of triglycerides was also measured using a blood assay. The reference range for triglycerides was 0–199 mg/dL. Hematocrit was measured within a reference range of 14.0–18.0 g/dL and 42–52%, respectively.

### Neuroimaging

#### Acquisition

A 3.0-T Siemens MAGNETOM Trio-Tim scanner was used to collect resting state scans using the following imaging parameters: TR = 1400 ms, TE = 30 ms, slice thickness = 3.0 mm, flip angle = 65°, field of view = 224 mm, slices = 64, and voxel size = 2.0 × 2.0 × 2.0 mm. Each participant’s acquisition time was 10 min using a multi-band imaging sequence. The subjects were instructed to lay still inside the scanner with their eyes open and were asked not to fall asleep. High-resolution anatomical images (MPRAGE) were acquired using the following scanning parameters: TR = 1900 ms, TE = 2.52 ms, slice thickness = 1.0 mm, flip angle = 9°, field of view = 256 mm, and voxel size = 1.0 × 1.0 × 1.0 mm. All fMRI data used in the analysis are part of the NKI Enhanced dataset made publicly available by the international neuroimaging data sharing initiative^33^. During their brain scanning session, physiological and resting state fMRI data were specifically collected and analyzed from the 1400 ms TR resting state session.

#### fMRI pre-processing

Resting state scans were preprocessed using DPARSF-A in DPABI (http://rfmri.org/DPARSF;^39,40^. The pipeline was implemented in MATLAB R2017a (MathWorks, Natick, MA, USA) using the following steps: (1) the first five images were removed; (2) nuisance covariates (Friston 24 motion parameters, white matter, & cerebrospinal fluid) and linear trends were regressed out; (3) band-pass filtering at 0.01 to 0.1 Hz^41^; (4) data were despiked through AFNI 3dDespike, realigned and normalized with DPARSF-A, and smoothed to 6 mm with AFNI 3dBlur; and (5) independent component analysis (ICA-FIX) was applied through FSL MELODIC to identify signal and noise components in individual subject space, which was subsequently extracted and transformed into 3 mm MNI-152 template space. Of the individuals from which MRI, TSC40, and GI self-report data were available, n = 69 were excluded due to excessive head motion, based on framewise displacement > 0.5 mm^42^.

#### Resting state functional connectivity analyses

We defined CEN regions of interest (ROIs) with a publicly available atlas^43^. For each of the CEN’s 10 ROIs^43^, we placed a 5-mm sphere around peak activation coordinates for each discrete cluster within the left- and right-hemisphere masks. Time-series data for each voxel were demeaned and converted to percent signal change scores to reduce variability between adults. We then calculated the ROI seed data as the average percent signal change for all voxels in each region. The rsFC was quantified as the Pearson correlation (r) relating the average time series in each ROI with the average time series in all other ROIs within the network. After converting these r values into Z-scores using Fisher’s transformation, we averaged all pairwise Z-scores within the network to form a summary statistic, reflecting the mean connectivity between all nodes within the CEN. We then performed a mean split of the average within-network rsFC of the CEN to form a group of high and low CEN rsFC, to test between-group factors as a main interaction effect in the model.

### Statistical analysis

The current analysis investigates the relationship between TSC-40 scores (independent variable), rsFC within the CEN (moderator), and GI burden (dependent variable). The primary objective is to determine if TSC-40 scores predict the number of endorsed GI-related issues and further explore the interaction between CEN connectivity and TSC-40 scores. In the primary model, raw summative TSC-40 scores were used. CEN connectivity is dummy coded (0 = low CEN connectivity, 1 = high CEN connectivity) based upon median split, and the number of endorsed GI-related issues was measured as count data.

Poisson regression models were specified with the primary study variables to assess their impact on the relationship between TSC-40 on GI burden as a function of CEN rsFC, given the count nature of the dependent variable. Subsequentially a secondary model was constructed to adjust for the potential demographic and cardiometabolic confounding effects. Researchers made a deliberate choice to incorporate cardiometabolic variables—specifically glucose, triglycerides, high-density lipoprotein (HDL), and low-density lipoprotein (LDL)—as covariates to mitigate potential confounding influences on the interplay between GI burden, TSC-40 scores, and resting-state functional connectivity (rsFC) within the Central Executive Network (CEN). This preemptive inclusion was guided by the known impact of metabolic health on neurocognitive functions^44,45^ and stress responses^46^, which are critical elements within the scope of our study.

Because of the potentially confounding effects of overlap in indicators of cardiometabolic status in the secondary model, multicollinearity will be rigorously assessed using the Variance Inflation Factor (VIF). A VIF threshold of 5 will be employed to indicate significant multicollinearity among the covariates. Should any of the covariates exhibit a VIF value equal to or exceeding this threshold, it will indicate a high degree of collinearity, which may necessitate further action. Possible steps to address identified multicollinearity agreed upon in the literature include examining for redundant variables, considering the removal or consolidation of highly collinear variables, or applying advanced statistical methods such as ridge regression. These approaches are intended to ensure that the model’s validity is not compromised by interdependencies among the predictors and that the interpretations of the regression coefficients remain reliable and meaningful. If necessary, overdispersion in the Poisson model will be checked, and a negative binomial regression will be considered. Assumptions of linearity, independence, and homoscedasticity will be verified. All analyses were conducted using R (R core Team, 2023) and various packages including dplyr^47^, psych^48^, ggplot^49^, and caret^50^.

## Results

A total of 378 individuals (ages 18–85.6 years, 64% female) with usable fMRI, behavioral, and self-report medical history data met the study inclusion criteria. A detailed comparison of demographics and medical history of persons in the low and high CEN connectivity groups is summarized in Table 1. Medical history encompassed a broad range of indicators of gastrointestinal and cardiometabolic disease. Notable differences were observed in age, with the low CEN group being older on average (51.47 ± 17.20) compared to the high CEN group (45.62 ± 18.39). TSC-40 scores, which reflect traumatic stress symptoms, were lower in the high CEN group (17.36 ± 12.44) than in the low CEN group (20.52 ± 12.45). In terms of racial composition, the percentage of individuals identified as Black was significantly higher in the high CEN group (24.43%) compared to the low CEN group (14.36%). Furthermore, the proportion of individuals diagnosed with IBS was higher in the low (13.86%) compared to the high CEN group (7.39%). Due to the dependent variable being count data, a Poisson regression was used to test the relationship between TSC-40 scores on the total number of endorsed gastrointestinal burden as a function of rsFC within the CEN.

**Figure 1:**
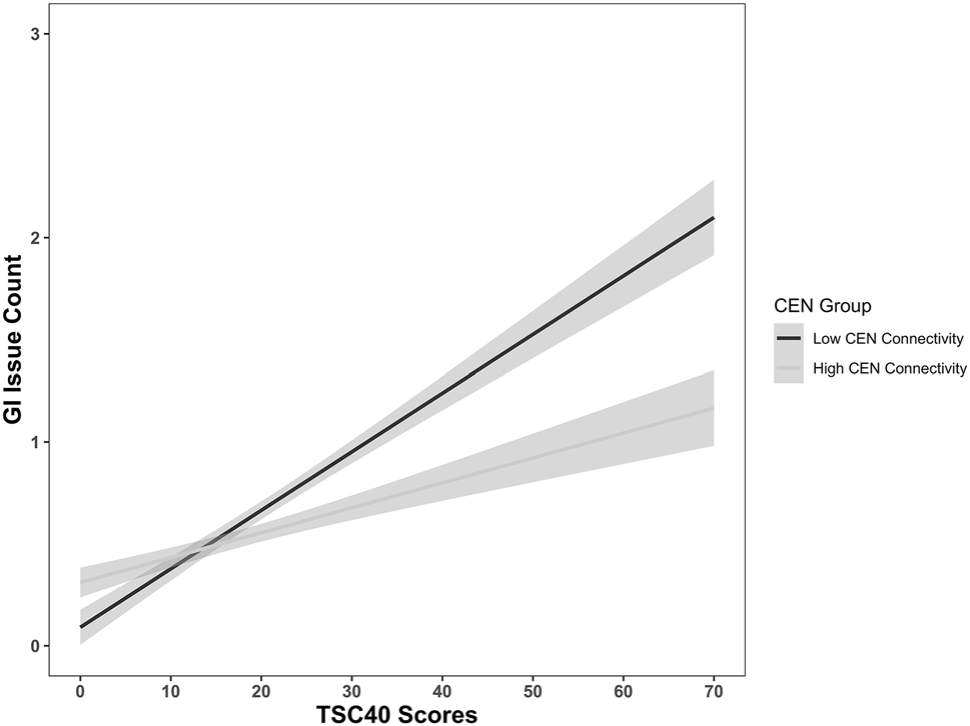
Endorsed gastrointestinal (GI) issue count and trauma symptom severity by central executive network (CEN) connectivity group. CEN connectivity (low vs. high) was found to be a moderator for the relationship between trauma symptom severity measured by the Trauma Symptom Severity Checklist (TSC-40) and the number of endorsed gastrointestinal issues (e.g., Irritable Bowel Syndrome, Chron’s Disease, Stomach/intestinal ulcers, general stomach/intestinal problems).

To examine the differential contributions of CEN group status (i.e., above or below rsFC within the CEN) on the relationship between TSC-40 on GI burden count, our adjusted model accounted for the following covariates: age, race, glucose, cholesterol, triglycerides, HDL, LDL, and hematocrit. In the unadjusted model, TSC-40 was positively associated with GI burden, *b* = −0.038, SE = 0.009, *p* < 0.001. Although there was no main effect for CEN group on GI burden count, the interaction term trended towards significance, *b* = −0.031, SE = 0.016, *p* 0.051. When controlling for covariates in the adjusted model, there is a main effect for CEN group (*b* = 0.659, SE = 0.278, *p* = 0.018), and the interaction term is significant (*b* = −0.030, SE = 0.010, *p* = 0.003) (Fig. 1). Among the listed covariates in the adjusted model, age (*b* = 0.022, SE = 0.004, *p* < 0.001) and race (*b* = 0.277, SE = 0.093, *p* = 0.003) were the only statistically significant covariates in the revised model (see Table 2). Post-hoc analysis using dummy coding for race and Black individuals as the reference group revealed that White individuals were more likely to endorse GI burden (*b* = 0.717, SE = 0.216, *p* < 0.001). This was the only racial group to significantly differ from the reference group.

**Table 2:** Regression table of the GI complain count model with or without covariates.

	Unadjusted model			Adjusted model		
	b	*p*	95% CI	b	*p*	95% CI
TSC × CEN	−0.018	0.051	[−0.036, −0.0001]	−0.029	0.004*	[−0.049, −0.009]
TSC-40 scores	0.038	< 0.001*	[0.027, 0.049]	0.047	< 0.001*	[0.034, 0.059]
CEN connectivity group	0.247	0.336	[−0.259, 0.750]	0.650	0.019*	[0.108, 1.190]
Age				0.022	< 0.001*	[0.013, 0.030]
Race				0.283	0.002*	[0.108, 0.474]
White				0.683	0.001*	[0.279, 1.122]
Native American				0.166	0.874	[−2.745, 1.802]
Asian				−0.934	0.066	[−2.114, −0.072]
Other				−0.310	0.667	[−2.119, 0.853]
Glucose				−0.001	0.888	[−0.007, 0.006]
Triglyceride				0.001	0.788	[−0.002, 0.002]
HDL				0.002	0.590	[−0.006, 0.010]
LDL				0.001	0.974	[−0.004, 0.004]

CI = confidence interval. Unadjusted model includes the neurological and psychological variables of interest and the interaction of the two variables. The adjusted model includes age, race, glucose, triglyceride, HDL, and LDL.

*p < .05.

## Discussion

This study aimed to test the assumption that PTS symptom severity is associated with GI issues and that rsFC within the CEN moderates the association between TSC-40 scores and total GI issues that are endorsed in the absence of a PTSD diagnosis. Consistent with previous literature, we observed a positive correlation between TSC-40 scores and the number of GI issues reported after controlling for demographic and cardiometabolic factors. In line with our prediction, this effect was moderated by rsFC within the CEN. That is, in individuals with above-average connectivity within the CEN, there was a significantly smaller effect of PTS symptoms on the number of endorsed GI disorders compared to those with below-average connectivity of that network. These findings suggest that synchronic activation of nodes within the CEN may support neurological processes that moderate the known effect of traumatic stress on downstream signaling pathways.

Previous work examines the effects of clinical PTSD on GI issues^16–18^ and implicates the CEN in PTSD and GI issues^31,32^. Our findings suggest that neurological mediating effects within the CEN on the relationship between PTS symptoms and the amount of endorsed GI-related issues can be observed in the absence of a clinical PTSD diagnosis. This highlights the need for a better understanding of the role of the CNS in the putative mechanisms linking traumatic stress exposure to the development of gastrointestinal disturbances in the context of executive functioning.

One such plausible neurobiological mechanism that has gained traction in recent years is the effect of the sympathoadrenal response on GI dysbiosis^18^. The sympathoadrenal system allows for the sympathetic nervous system to elicit whole-body responses to stress via the adrenal medulla stimulation. Given the role of norepinephrine (NE) as a neuroendocrine molecule that interfaces central, autonomic, and enteric nervous systems, interest in the modulatory role of this neuroendocrine molecule in neuroimmune signaling in PTSD has risen. A recent meta-analysis of over 27 studies of combat and non-combat-related PTSD revealed significantly elevated levels of NE, but not cortisol or epinephrine when compared to non-traumatized controls^51^. As alluded to, the support for elevated NE in persons experiencing PTS dovetails with the current zeitgeist surrounding the stress-diathesis model of gut-brain axis interactions, wherein increased adrenergic signaling precipitates gut pathogenesis, including cell-mediated inflammation, altered gut motility and permeability, and proliferation of non-commensal gut flora^1,52–55^.

Given the robust association between stress-related NE efflux and gut dysbiosis, our findings beg the question of exactly how rsFC of the CEN may mitigate NE expression in the process. Several research groups have turned their attention to the locus coeruleus (LC) as a potential target for neuroimmune signaling implicated in the pathophysiology of PTS. As the primary producer of NE, neurons within this small region of the brainstem influence the arousal state^56,57^. The CEN maintains inhibitory and excitatory control over the arousal state through projections to the NE neurons in the LC^58^. In a recent study, stress-related LC activity resulting from a serial response inhibition task was mediated by functional connectivity within prefrontal regions, namely the inferior frontal gyrus^55^. More sophisticated task-based research paradigms are required to determine whether synchrony within the CEN during real-time exposure to stress mitigates LC activity and downstream objective markers of gut dysbiosis.

Although this study focused on how rsFC within the CEN moderates the relationship between PTS and GI issues, additional psycho-neuro-immune research is needed to determine whether this mechanism extends to other allostatic disease processes. For example, a longitudinal study comparing two-year changes in CEN connectivity among children exposed to neighborhood violence found that higher rsFC of the CEN mitigated the effect of stress exposure on lipopolysaccharide-stimulated peripheral blood mononuclear cell expression of pro-inflammatory cytokines^59^. Given that gut dysbiosis shares pathophysiological pathways and is associated with a myriad of poor health outcomes, including increased susceptibility to infections^60^, cardiovascular disease and obesity^61^, certain cancers^62^, and psychiatric disorders^63–65^, more work is needed to understand how the organization of the CEN may protect against or exacerbate stress-related diseases.

Previous literature has also shown that psychosocial stress plays a role in certain GI disorders^66^. Racially/ethnically minoritized groups are known to encounter more psychosocial challenges in their daily lives than their non-minoritized counterparts^67^. In our adjusted model, we used race as a covariate for GI count and found that White individuals were significantly more likely to endorse GI issues than Black individuals. This is in line with other literature that analyzed the prevalence of self-reported GI issues in the context of race. However, less is known about the underlying reasons for the disparities in self-reported GI issues. More research must assess if such discrepancies are due to underreporting, access, resilience, or a difference in GI-related disease prevalence.

### Limitations and future directions

Despite the compelling findings of this study, it is important to acknowledge several limitations that might impact the generalizability and interpretation of our results.

Firstly, the sample lacks ethnic diversity. The sample’s composition was predominantly white and notably excluded Hispanic individuals. The demographics of this study present a limitation in reflecting the full spectrum of racial and ethnic experiences, particularly given the documented disparities in health outcomes, including PTSD^68^, GI issues^69^, and brain connectivity^70^ across different ethnic groups. The over-representation of white participants risks biasing the results towards their experiences, potentially obscuring critical variations in how different racial and ethnic groups experience or express the phenomena within this study. Such limitations not only narrow our understanding but may also perpetuate existing health disparities by failing to adequately capture and analyze the unique health profiles and social experiences of more diverse populations. Future research must strive for greater racial and ethnic diversity, including refined categorizations of race/ethnicity (e.g., Middle Eastern/North Africans are often conflated as White) to broaden the applicability and enrich the relevance of the findings.

Secondly, the incidence of GI issues in this sample was based on self-report. Moreover, information on the history, duration, and severity of GI diagnoses endorsed was not collected or verified. Future models should be able to account for GI disease history as potential moderators of the observed relationships. Such information can further help to elucidate the pathophysiological mechanisms involved in stress-related GI disease processes.

Finally, this study was cross-sectional in nature, and the gut-brain axis is a well-documented bidirectional pathway, further limiting causal inferences drawn regarding the effect of PTS on GI issues. This design restricted our ability to establish temporal precedence, a crucial element for causal claims. Without establishing temporal precedence our data may also speak to the contribution of GI disease comorbidity to the exacerbation of PTS symptom severity. Future longitudinal investigations may help to determine whether the incidence of GI disease or connectivity within the CEN predicts the progression of PTS to a clinical diagnosis of PTSD.

A potential future direction of research is through PTSD treatments in trauma-exposed subclinical populations, as empirical evidence supports the plasticity of the rsFC as a potential therapeutic mechanism. For example, cognitive processing therapy (CPT) was found to normalize CEN connectivity following 12 sessions of treatment within a cohort of traumatized women^71^. Furthermore, CPT increased global connectivity in the CEN in veterans with PTSD^72^. Cognitive behavioral therapy has also demonstrated similar results for PTSD, with 12 weeks of manualized sessions significantly increasing intrinsic functional connectivity between the amygdala and regions within the CEN^73^. Another cognitive-oriented therapy (mindfulness-based exposure therapy) has similarly resulted in an increase in connectivity between prefrontal structures, along with reductions in avoidance and hyperarousal symptomology, among veterans with PTSD^74^. Given this evidence across an array of therapeutic approaches, it appears that CEN connectivity covaries as a function of trauma symptom severity and, therefore, may mitigate the extent to which the processing of trauma-related stressors impacts physiological arousal and its outcomes. While we were not privy to the types of traumatic events experienced by participants or when that experience took place, these are important characteristics that should be considered in future work as they might mitigate therapeutic effects.

Nonetheless, there is parallel evidence supporting the impact of cognitive emotion regulation strategies on gastrointestinal complaints is robust^75–78^. A systematic review on the efficacy of cognitive behavioral therapy for IBS demonstrated that it is moderately effective across various formats of administration, and the benefits are sustained through long-term follow-up^79^. Interestingly, a randomized controlled trial implementing this form of therapy for persons with IBS demonstrated improvements in GI symptomology, independent of its effects on psychological distress^77^.

## Conclusion

To our knowledge, this is the first paper to directly assess the extent to which CEN connectivity moderates the relationship between trauma symptom severity and GI issues in a non-clinical PTSD sample. Moving forward understanding the underlying gut-brain axis mechanisms may not only deepen our insights into the role of frontal lobe functioning in buffering stress-related GI diseases but also pave the way for novel therapeutic interventions. Moreover, this and other investigations support the use of cognitive and behavioral strategies to address post-traumatic stress-related GI issues, by enhancing functional connectivity of brain regions supporting executive functioning. Thus, it is imperative to foster an integrated approach to bridging the gap between neuroscience, psychology, and gastroenterology in order to facilitate mental and physical well-being.

## Data Availability

The datasets used and/or analyzed during the current study available from the corresponding author on reasonable request.
